# Synthesis of 6-PEt*N*-α-D-Gal*p*NAc-(1–>6)-β-D-Gal*p*-(1–>4)-β-D-Glc*p*NAc-(1–>3)-β-D-Gal*p*-(1–>4)-β-D-Glc*p*, a *Haemophilus influenzae* lipopolysacharide structure, and biotin and protein conjugates thereof

**DOI:** 10.3762/bjoc.6.80

**Published:** 2010-07-26

**Authors:** Andreas Sundgren, Martina Lahmann, Stefan Oscarson

**Affiliations:** 1 Department of Chemistry, Göteborg University, S-412 96 Gothenburg, Sweden; 2 The School of Chemistry, University of Bangor, Alun Roberts Building, Deiniol Road, Bangor, Gwynedd LL57 2UW, U.K.; 3 Centre for Synthesis and Chemical Biology, University College Dublin, Belfield, Dublin 4, Ireland, Phone: +353 17162318

**Keywords:** conjugate vaccines, glycoconjugates, *Haemophilus influenzae*, lacto-N-neotetraose, oligosaccharide synthesis, thioglycosides

## Abstract

**Background:** In bacteria with truncated lipopolysaccharide structures, i.e., lacking the O-antigen polysaccharide part, core structures are exposed to the immune system upon infection and thus their use as carbohydrate surface antigens in glycoconjugate vaccines can be considered and investigated. One such suggested structure from *Haemophilus influenzae* LPS is the phosphorylated pentasaccharide 6-PEt*N*-α-D-Gal*p*NAc-(1→6)-β-D-Gal*p*-(1→4)-β-D-Glc*p*NAc-(1→3)-β-D-Gal*p*-(1→4)-β-D-Glc*p*.

**Results:** Starting from a spacer-containing lactose derivative a suitably protected lacto-*N*-neotetraose tetrasaccharide structure was constructed through subsequential couplings with two thioglycoside donors, a glucosamine residue followed by a galactose derivative, using NIS/AgOTf as promoter. Removal of a silyl protecting group at the primary position of the non-reducing end residue afforded an acceptor to which the terminal α-galactosamine moiety was introduced using a 2-azido bromo sugar and halide assisted coupling conditions. Global deprotection afforded the non-phosphorylated target pentasaccharide, whereas removal of a silyl group from the primary position of the non-reducing end residue produced a free hydroxy group which was phosphorylated using H-phosphonate chemistry to yield the phosphoethanolamine-containing protected pentasaccharide. Partial deprotection afforded the phosphorylated target pentasaccharide with a free spacer amino group but with a protected phosphoethanolamino group. Conjugation of the spacer amino group to biotin or dimethyl squarate followed by deprotection of the phosphoethanolamino group and, in the case of the squarate derivative, further reaction with a protein then afforded the title conjugates.

**Conclusion:** An effective synthesis of a biologically interesting pentasaccharide structure has been accomplished. The target pentasaccharide, an α-GalNAc substituted lacto-*N*-neotetraose structure, comprises a phosphoethanolamine motif and a spacer aglycon. Through the spacer, biotin and protein conjugates of the title compound have been constructed to allow further use in biological experiments.

## Introduction

**Figure 1:**
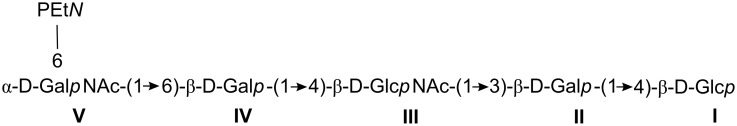
The *H. influenzae* outer core target structure.

*Haemophilus influenzae* are Gram-negative bacteria divided into six serotypes, a–f, related to the structure of the capsular polysaccharide usually surrounding the bacterium [1]. Among these serotypes, type b is the cause of the most severe diseases, i.a. meningitis and pneumonia. However, there are now several commercial vaccines against this serotype that have proven to be highly effective [2]. These vaccines are glycoconjugate vaccines, based on capsular poly- or oligosaccharide structures, either native or synthetic [3–4], linked to a carrier protein. The lipopolysaccharide (LPS) of *H. influenzae* shows a huge structural variety and hence non-capsulated bacteria are referred to as non-typable *H. influenzae* (NTHi) [5–6]. This structural diversity is a good defence mechanism against the human acquired immune system, and NTHi often cause repetitive infections. Another way used by *H. influenzae* to avoid the human immune system seems to be molecular mimicry, i.e. the bacteria express common human carbohydrate structures on their surface that the host recognises as self-structures and do not produce antibodies against. Construction of a glycoconjugate vaccine against NTHi is thus quite complex. Carbohydrate structures that are both exposed on the surface of most bacteria and also immunogenic in humans must be found and produced. To tackle this problem we are presently pursuing two possible routes. A common conserved LPS inner-core pentasaccharide structure has been identified, and efforts to produce this structure, i.a. through synthesis, are on-going [7–9]. Furthermore, analysis of the LPS of NTHi strains to find frequent non-human outer-core structures is continuously performed [5–6]. One candidate recently suggested is a lacto-*N*-*neo*tetraose structure substituted with a PEt*N*-GalNAc residue (Figure 1) [10]. Herein we describe the synthesis of this structure and its conjugation to biotin and a carrier protein to form glycoconjugates that can be used in biological experiments to evaluate the immunological properties of the title structure.

## Results and Discussion

**Scheme 1:**
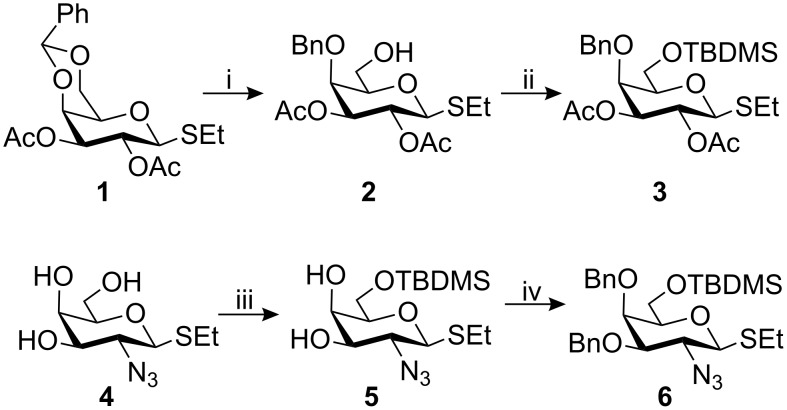
i. BH_3_, Bu_2_BOTf, THF/CH_2_Cl_2_, 85%; ii. TBDMSCl, pyridine, CH_2_Cl_2_, 90%; iii. TBDMSCl, pyridine, 92%; iv. BnBr, NaH, DMF, 85%.

The target structure is, as noted above, a substituted lacto-*N*-neotetraose structure that we have experience of from, i.a., former synthetic work related to *Streptococcus pneumoniae* type 14 CPS structures [11]. To allow for the introduction of the GalNAc residue at the 6^IV^-position and the subsequent phosphorylation of the 6^V^-hydroxy group, two new galactosyl donors were designed and synthesised (Scheme 1). Reductive ring opening, with BH_3_/Bu_2_BOTf [12], of the benzylidene acetal in the known ethyl thioglycoside **1** [13] gave the 6-hydroxy derivative **2** (85%), which was then silylated to afford donor **3** (90%). Regioselective silylation of the 2-azido-galactose ethyl thioglycoside **4** [14] yielded the 6-*O*-silylated compound **5** (92%), benzylation of which gave donor **6** (85%). A benzyl group in the 4-position was preferred to an ester group to avoid the risk of acyl migration during subsequent reactions; desilylation, glycosidation and phosphorylation.

**Scheme 2:**
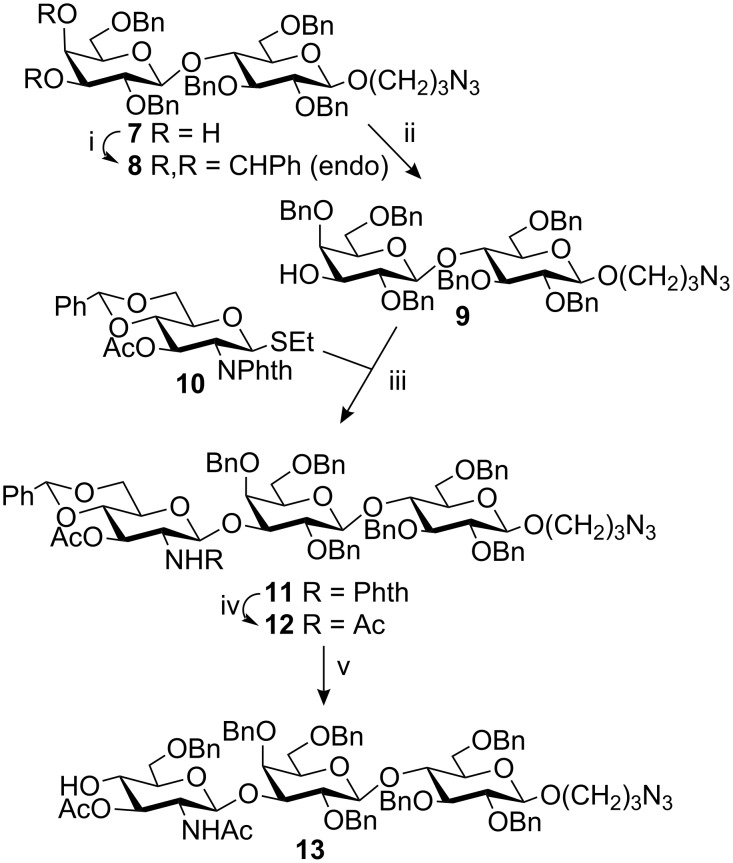
i. PhCH(OMe)_2_, CSA; ii. NaBH_3_CN, HCl/Et_2_O, THF, 80%; iii. NIS/AgOTf, CH_2_Cl_2_, 83%; iv. a) NaOMe, MeOH; b) NH_2_(CH_2_)_2_NH_2_, EtOH; c) Ac_2_O, pyridine, 93%; v. NaBH_3_CN, HCl/Et_2_O, THF, 81%.

3^II^,4^II^-Diols of lactose are often used as acceptors for regioselective glycosidations in the 3-position. However, the selectivity is dependent on the donor, promoter and conditions employed [15], e.g., it has been shown that donors containing a 4,6-*O*-benzylidene acetal can give a mixture of products [16]. Hence, the diol **7** [17] was transformed using benzaldehyde dimethyl acetal and camphorsulfonic acid stereo-selectively into the corresponding 3,4-*endo*-benzylidene derivative **8** which was in turn converted to the 3-hydroxy derivative **9** by NaBH_3_CN/HCl-mediated reductive opening of the acetal ring [18] (80% overall yield from **7**, Scheme 2). NIS/AgOTf-promoted glycosidation of this acceptor with donor **10** [19] (1.4 equiv) then efficiently gave the β-linked trisaccharide **11** (83%). At this stage the phthalimido group was removed by aminolysis and the resulting amino compound acetylated to yield **12** (93%) with the target acetamido function in place. Once more reductive opening of a benzylidene acetal (NaBH_3_CN/HCl) gave a new mono-hydroxy compound, the acceptor **13** (81%).

**Scheme 3:**
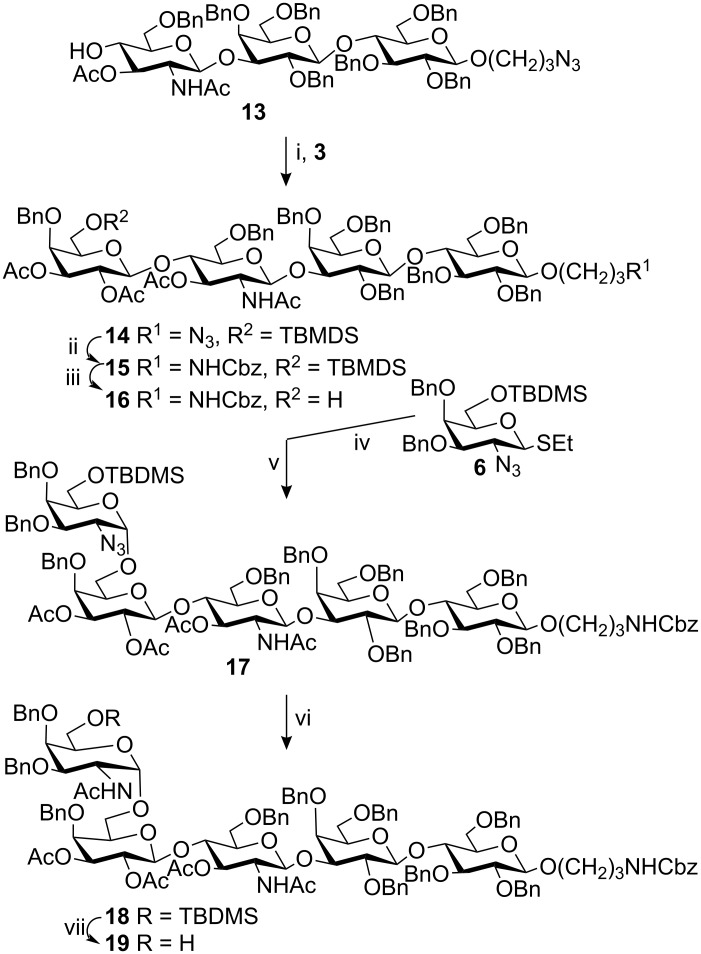
i. NIS/AgOTf, CH_2_Cl_2_, 77%; ii. a) H_2_S, pyridine, Et_3_N; b) CbzCl, pyridine, CH_2_Cl_2_, 91%; iii. TBAF, THF, 89%; iv. Br_2_, CH_2_Cl_2_; v. Et_4_NBr, DMF, CH_2_Cl_2_, 79%; vi. a) H_2_S, pyridine, Et_3_N; b) Ac_2_O, pyridine, CH_2_Cl_2_, 91%; vii. TBAF, THF, 85%.

The 4-hydroxy group in GlcNAc derivatives is known to be quite unreactive towards glycosylations, which, i.a., has led to development of new protecting group patterns to improve the reactivity [20–21]. Here, however, NIS/AgOTf-promoted glycosylation of acceptor **13** with donor **3** proceeded without difficulties to produce the tetrasaccharide **14** in high yield (77%, Scheme 3). Before the introduction of the azido-galactose residue, the spacer azido function was reduced to an amino group, which was protected as a benzyl carbamate (→ **15**, 91%). Removal of the TBDMS group with TBAF then gave the tetrasaccharide acceptor **16** (89%). To obtain complete α-selectivity with 2-azido-galactose donors is not trivial. A 4,6-silyl acetal has been suggested as one way to improve the selectivity [22]. We have earlier tried halide-assisted conditions [23], which is not that common due to the incompatibility between the mild coupling conditions and the low reactivity of 2-azido donors, with good results when simple spacer alcohols were used as aglycons [24]. This approach also worked well here, although extended reaction time was necessary. A halide-assisted coupling between acceptor **16** and the bromosugar, obtained from thioglycoside **6**, gave (after 11 days) the α-linked pentasaccharide **17** in 79% yield. Due to the excess of donor used and the long reaction time required the acetamido carbonyl oxygen also partly behaved as a nucleophile and gave a pseudohexasaccharide acetimidate side product according to MALDI-TOF and NMR. Similar side products have earlier been described [25–28]. Mild acid treatment of the glycosylation mixture prior to purification led to cleavage of the formed imidate and afforded the desired pentasaccharide in the above noted yield.

**Scheme 4:**
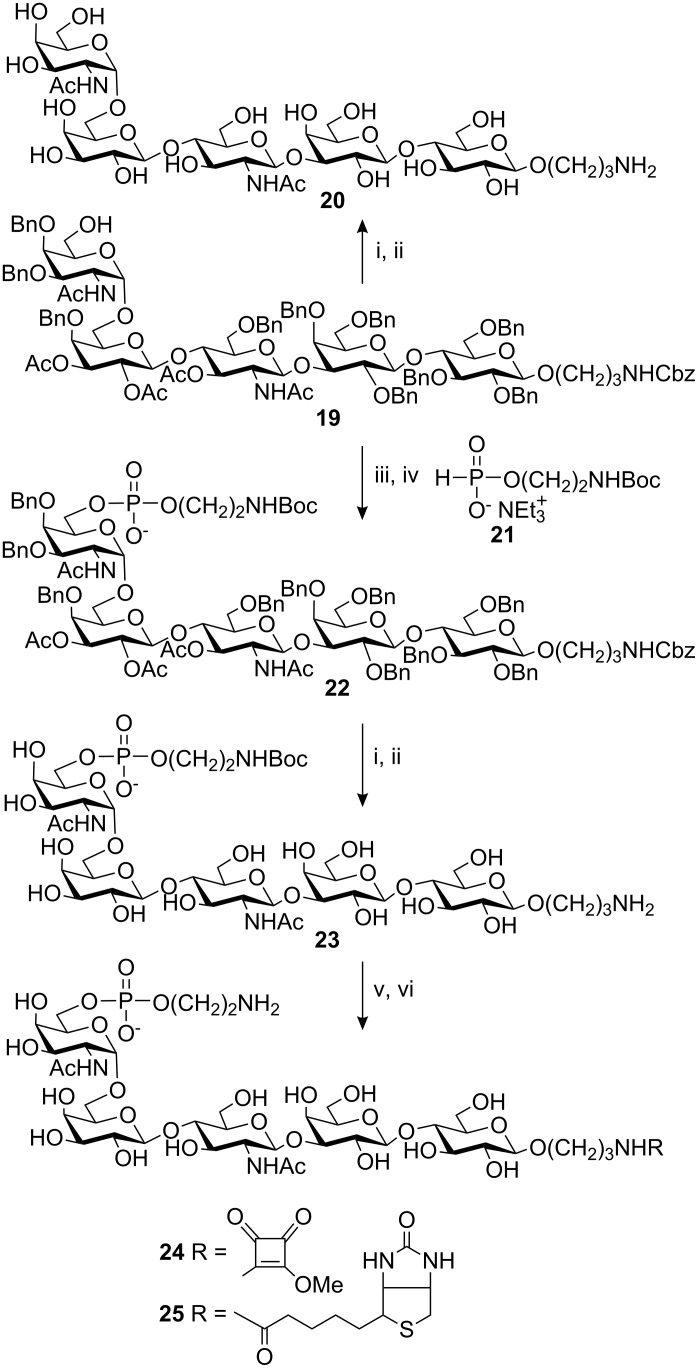
i. NaOMe, MeOH; ii. H_2_, Pd/C, MeOH/H_2_O; iii. **21**, PivCl, pyridine, MeCN; iv. I_2_, H_2_O, pyridine; v. Dimethyl squarate or NHS-biotin, buffer pH 7; vi. TFA (10% aqueous).

Reduction of the azido group in **17** and subsequent acetylation afforded compound **18** (91%). Removal of the TBDMS group, again using TBAF, gave derivative **19** (85%), ready for the introduction of the phosphoethanolamine. Compound **19** was also completely deprotected by sodium methoxide treatment followed by catalytic hydrogenolysis to give the non-phosphorylated target structure **20** (66%, Scheme 4), to be used as a reference in biological experiments. Earlier we used the Cbz-protected ethanolamine H-phosphonate monoester as a reagent in the formation of phosphoethanolamines [29]. Since the amino group in the spacer was already Cbz-protected and we wanted to be able to differentiate between the two amino groups during conjugation, a Boc-protected H-phosphonate monoester **21** was synthesised and used in the phosphorylation step. Activation of **21** with pivaloyl chloride in the presence of **19** afforded the H-phosphonate diester, which was oxidised with I_2_/pyridine in water to afford the phosphate diester **22** [30]. Deprotection with sodium methoxide followed by catalytic hydrogenolysis then afforded the still Boc-protected phosphoethanolamine pentasaccharide **23** (44% from **19**) ready for conjugation via the free spacer amino group.

The protein conjugations were carried out using squarate ester methodology [31–32]. Reaction of compound **23** with dimethyl squarate at neutral pH afforded the monomethyl ester squarate amide of **23**, from which the Boc-group was removed by acid hydrolysis to afford derivative **24**. Protein conjugations were performed by reaction of **24** (20 equiv) with human serum albumin (HSA). Compound **20** was similarly activated with dimethyl squarate and conjugated to HSA. MALDI-TOF MS of the HSA-conjugates of compounds **20** and **24** showed a loading of 16 oligosaccharides/protein molecule and 7 oligosaccharides/protein molecule, respectively. Since derivative **24** contains a free amino group there is a possibility of oligomerisation in addition to protein conjugation. To what extent this happened was not examined. Compound **23** was also biotinylated for use in ELISA-screening of antibodies raised against native LPS. Reaction with the commercial NHS-activated ester of biotin followed by TFA-treatment to remove the Boc-group afforded derivative **25**.

## Supporting Information

File 1 Experimental Section
