# Supplementary material for: Synthesis of 6-PEtN-α-D-GalpNAc-(1–>6)-β-D-Galp-(1–>4)-β-D-GlcpNAc-(1–>3)-β-D-Galp-(1–>4)-β-D-Glcp, a Haemophilus influenzae lipopolysacharide structure, and biotin and protein conjugates thereof
Source: Beilstein J Org Chem. 2010 Jul 26;6:704–8. doi: 10.3762/bjoc.6.80 (PMC2956385; doi:10.3762/bjoc.6.80)
Supplement: File 1 — Experimental Section [file Beilstein_J_Org_Chem-06-704-s001.pdf]

Supporting Information

for

Synthesis of 6-PEt*N*-α-D-Gal*p*NAc-(1→6)-β-D-Gal*p*-(1→4)-β-D-Glc*p*NAc-(1→3)-β-D-Gal*p*-(1→4)-β-D-Glc*p*, a *Haemophilus influenzae* lipopolysacharide structure, and biotin and protein conjugates thereof

Andreas Sundgren[1], Martina Lahmann[2] and Stefan Oscarson*[3]

[1]Department of Chemistry, Göteborg University, S-412 96 Gothenburg, Sweden; [2]The School of Chemistry, University of Bangor, Alun Roberts Building, Deiniol Road, Bangor, Gwynedd LL57 2UW, U.K. and [3]Centre for Synthesis and Chemical Biology, University College Dublin, Belfield, Dublin 4, Ireland, Phone: +353 17162318

E-Mail: Andreas Sundgren - andreas@riktad.com; Martina Lahmann - m.lahmann@bangor.ac.uk; Stefan Oscarson - stefan.oscarson@ucd.ie

*Corresponding author

General methods

Organic solutions were dried over $MgSO_4$ or Na_2SO_4 before concentration, which was performed under reduced pressure at <45 °C (bath temperature). NMR spectra were recorded at 25 °C and 400 MHz (^{1}H) in $CDCl_3$ with Me_4Si as internal standard (δ = 0.00), 75 or 100 MHz (^{13}C) in $CDCl_3$ with $CDCl_3$ as internal standard (δ = 77.16) or 67 MHz (^{31}P) in H_2O with H_3PO_4 as external standard (δ = 0.00), unless otherwise stated. TLC was performed on silica gel 60 F254 with detection by charring with 8% sulfuric acid or ninhydrin. Silica gel (0.040–0.063 mm) was used for column chromatography.

Ethyl 2,3-di-*O*-acetyl-4-*O*-benzyl-6-*O*-*tert*-butyldimethylsilyl-1-thio-β-D-galactopyranoside (3). Ethyl 2,3-di-*O*-acetyl-4,6-*O*-benzylidene-1-thio-β-D-galactopyranoside (**1**, 1.43 g, 3.61 mmol) [13] was dissolved in 1M BH_3 in THF (36 mL) and 1M Bu_2BOTf in CH_2Cl_2 (4.1 mL) was added slowly at 0 °C. After 2 h, triethylamine (2 mL) was added followed by slow addition of MeOH (10 mL). The solution was concentrated, coevaporated with MeOH three times and with toluene two times, and the residue purified by silica gel column chromatography (toluene-EtOAc 2:1) to yield ethyl 2,3-di-*O*-acetyl-4-*O*-benzyl-1-thio-β-D-galactopyranoside (**2**, 1.22 g, 3.07 mmol, 85%). ^{13}C NMR ($CDCl_3$): δ 20.8, 20.8 ($COCH_3$), 61.4, 68.1, 74.0, 74.9, 74.9, 78.0, 83.5 (C-1-C-6, CH_2Ph), 127.8–138.0 (aromatic *C*), 169.6, 170.3 ($COCH_3$). A solution of TBDMS-Cl (633 mg, 4.22 mmol), pyridine (1.0 mL) and **2** (1.02 g, 2.56 mmol) in CH_2Cl_2 (15 mL) was stirred for 18 h. MeOH (1.0 mL) was added and the solution concentrated, coevaporated with toluene and the residue purified by column chromatography (toluene-EtOAc 3:1) to give **3** (1.18 g, 2.30 mmol, 90%). $[\alpha]_D$ +4.4 (*c* 1.1, $CHCl_3$). ^1H NMR ($CDCl_3$): δ 0.03 (s, 6H, $SiCH_3$), 0.87, 0.88 (s, 9H, $C(CH_3)_3$), 1.23 (t, 3H, SCH_2CH_3), 1.93, 2.04 (s, 6H, $COCH_3$), 2.70 (m, 2H, SCH_2CH_3), 3.58 (t, 1H, H-5), 3.72–3.74 (m, 2H, H-6), 4.04 (d, 1H, H-4), 4.40 (d, 1H, $J_{1,2}$ 9.88 Hz, H-1), 4.61, 4.70 (d, 2H, CH_2Ph), 4.99 (dd, 1H, H-3), 5.39 (t, 1H, H-2), 7.25–7.34 (m, 5H, aromatic *H*). ^{13}C NMR ($CDCl_3$): δ −5.4, −5.3 ($SiCH_3$), 14.9 (SCH_2CH_3), 18.3 ($C(CH_3)_3$), 20.9, 21.0 ($COCH_3$), 23.6 (SCH_2CH_3), 26.0 ($C(CH_3)_3$), 60.9, 68.3, 74.2, 75.0, 75.1, 78.9 (C-2-6, CH_2Ph), 83.5 (C-1), 127.9–138.1 (aromatic *C*), 169.8, 170.1 ($COCH_3$). Anal. Calcd for $C_{25}H_{40}O_7SSi$: C, 58.56; H, 7.86. Found: C, 58.46; H, 7.92.

Ethyl 2-azido-3,4-di-*O*-benzyl-2-deoxy-6-*O*-*tert*-butyldimethylsilyl-1-thio-β-D-galactopyranoside (6). Pyridine (1.2 mL) and TBDMS-Cl (1.69 g, 6.15 mmol) were added to a solution of 2-azido-2-deoxy-1-thio-β-D-galactopyranoside (**4**, 1.02 g, 4.10 mmol) [14] in pyridine (17 mL) and the reaction was stirred for 48 h. MeOH (5 mL) was added and the solution concentrated, coevaporated with toluene and the residue purified by column chromatography (toluene-EtOAc 2:1) to obtain ethyl 2-azido-2-deoxy-6-*O*-*tert*-butyldimethylsilyl-1-thio-β-D-galactopyranoside (**5**, 1.86 g, 3.79 mmol, 92%). $[\alpha]_D$ +7.6° (*c* 1.0, $CHCl_3$). ^{1}H NMR ($CDCl_3$): δ 1.07 (s, 9H, $C(CH_3)_3$), 1.30 (t, 3H, SCH_2CH_3), 2.74 (m, 2H, SCH_2CH_3), 3.46 (t, 1H, H-5), 3.51 (m, 1H, H-3), 3.60 (t, 1H, H-2), 3.91 (d, 2H, H-6), 4.10 (s, 1H, H-4), 4.26 (d, 1H, H-1), 7.40–7.73 (m, 10H, aromatic *H*). ^{13}C NMR ($CDCl_3$): δ 15.1 (SCH_2CH_3), 19.2 ($C(CH_3)_3$), 24.4 (SCH_2CH_3), 26.9 ($C(CH_3)_3$), 63.8, 63.8, 69.0, 74.4, 77.8 (C-2-6), 84.3 (C-1), 127.9–135.8 (aromatic *C*). Compound **5** (1.86 g, 3.79 mmol) was dissolved in DMF (15 mL) and 60% NaH (661 mg, 16.5 mmol) added. After stirring for 10 min, benzyl bromide (1.50 mL, 12.6 mmol) was added, the reaction stirred for 30 min followed by slow addition of MeOH (4 mL). The mixture was diluted with toluene, washed with water (2 × 15 mL), concentrated and the residue coevaporated with toluene. Purification by column chromatography (pentane → pentane-toluene 1:10) gave **6** (2.16 g, 3.24 mmol, 85%). $[\alpha]_D$ −0.4 (*c* 1.7, $CHCl_3$). ^{1}H NMR ($CDCl_3$): δ 1.10 (s, 9H, $C(CH_3)_3$), 1.29 (t, 3H, SCH_2CH_3), 2.72 (m, 2H, SCH_2CH_3), 3.41–3.47 (m, 2H, H-3, H-5), 3.83 (d, 2H, H-6), 3.90 (t, 1H, H-2), 4.01 (d, 1H, H-4), 4.23 (d, 1H, H-1), 4.64 (d, 1H, CH_2Ph), 4.80 (d, 2H, CH_2Ph), 4.97 (d, 1H, CH_2Ph), 7.28–7.68 (m, 20H, aromatic *H*). ^{13}C NMR ($CDCl_3$): δ 15.1 (SCH_2CH_3), 19.3 ($C(CH_3)_3$), 24.3 (SCH_2CH_3), 27.0 ($C(CH_3)_3$), 62.3, 62.8, 72.4, 72.7, 74.7, 78.9, 82.7, 84.2 (C-1-6, CH_2Ph), 127.6–138.6

(aromatic *C*). Anal. Calcd for $C_{38}H_{45}N_3O_4SSi$: C, 68.33; H, 6.79. Found: C, 68.42; H, 6.68.

3-Azidopropyl (3-*O*-acetyl-4,6-*O*-benzylidene-2-deoxy-2-phthalimido-β-D-glucopyranosyl)-(1→3)-(2,4,6-tri-*O*-benzyl-β-D-galactopyranosyl)-(1→4)-2,3,6-tri-*O*-benzyl-β-D-glucopyranoside (11). CSA (90 mg) was added to a solution of 3-azidopropyl (2,6-di-*O*-benzyl-β-D-galactopyranosyl)-(1→4)-2,3,6-tri-*O*-benzyl-β-D-glucopyranoside (**7**, 1.46 g, 1.67 mmol) [17] and benzaldehyde dimethyl acetal (490 μL, 3.26 mmol) in DMF (15 mL). After stirring at 40 °C under reduced pressure for 1.5 h, triethylamine (2 mL) and toluene (25 mL) was added. The solution was washed with water and $NaHCO_3$ (aq), dried, concentrated and purified by column chromatography (toluene-EtOAc 18:1) to give 3-azidopropyl (2,6-di-*O*-benzyl-3,4-*O*-*endo*-benzylidene-β-D-galactopyranosyl)-(1→4)-2,3,6-tri-*O*-benzyl-β-D-glucopyranoside (**8**)**.** The residue from last step and $NaBH_3CN$ (890 mg, 13.5 mmol) in dry THF (20 mL) containing 3Å molecular sieves was stirred for 10 min followed by slow addition of HCl/Et_2O (sat.) until no starting material could be detected by TLC. The mixture was diluted with toluene (30 mL), filtered through Celite, washed with water and stirred with 0.1 M HCl (20 mL, aq) overnight. The organic layer was washed with water, $NaHCO_3$ (aq), dried, concentrated and purified by column chromatography (toluene-EtOAc 9:1) to yield 3-azidopropyl (2,4,6-tri-*O*-benzyl-β-D-galactopyranosyl)-(1→4)-2,3,6-tri-*O*-benzyl-β-D-glucopyranoside (**9**, 1.28 g, 1.33 mmol, 80%). ^{13}C NMR ($CDCl_3$): δ 29.4 ($OCH_2\mathit{C}H_2CH_2N_3$), 48.4 ($OCH_2CH_2\mathit{C}H_2N_3$), 66.6, 68.1, 68.3, 68.5, 73.3, 73.4, 73.5, 73.6, 74.2, 75.1, 75.2, 75.5, 76.0, 76.7, 80.7, 81.9, 83.0 (C-2^{I}-6^{I}, 2^{II}-6^{II}, $O\mathit{C}H_2CH_2CH_2N_3$, $\mathit{C}H_2Ph$), 102.8, 103.6 (C-1^{I}, 1^{II}), 126.5–

139.2 (aromatic *C*). Ethyl 3-*O*-acetyl-4,6-*O*-benzylidene-2-deoxy-2-phthalimido-1-thio-β-D-glucopyranoside (**10**, 3.21 g, 6.65 mmol) [19] and **9** (6.74 g, 4.86 mmol) were dissolved in dry CH_2Cl_2 (30 mL) and 4Å molecular sieves was added. The mixture was stirred for 30 min, cooled to −40 °C followed by addition of NIS (3.01 g, 13.4 mmol). After another 20 min AgOTf (120 mg) was added and the reaction mixture stirred for 35 min at −25 °C, quenched with Et_3N, diluted with toluene, filtered through Celite, concentrated and purified by column chromatography (toluene-EtOAc 6:1) to afford **11** (5.59 g, 4,03 mmol, 83%). $[\alpha]_D$ −42.4 (*c* 0.5, $CHCl_3$). ^{13}C NMR ($CDCl_3$): 20.6, 20.8 ($COCH_3$), 29.3 ($OCH_2CH_2CH_2N_3$), 48.4 ($OCH_2CH_2CH_2N_3$), 55.4, 55.5, 65.9, 66.1, 66.4, 66.5, 67.7, 67.8, 68.0, 68.3, 68.4, 69.3, 69.5, 69.7, 70.6, 72.2, 72.5, 73.2, 73.2, 73.5, 74.1, 74.1, 74.3, 74.6, 74.8, 75.1, 75.1, 75.4, 75.6, 75.8, 75.8, 77.3, 77.9 ,78.1, 79.0, 79.8, 81.6, 81.8, 82.9, 83.1, 85.5 (C-2^{I}-6^{I}, 2^{II}-6^{II}, 2^{III}-6^{III}, $OCH_2CH_2CH_2N_3$, CH_2Ph), 100.1, 101.8, 102.4, 103.5 (C-1^{I}, 1^{II}, 1^{III}, *C*HPh), 125.5–138.6 (aromatic *C*), 167.1, 168.5, 170.4 ($COCH_3$, Phth*C*O). Anal. Calcd for $C_{74}H_{82}N_4O_{17}$: C, 69.25; H, 5.96. Found: C, 69.18; H, 6.10.

3-Azidopropyl (2-acetamido-3-*O*-acetyl-6-*O*-benzyl-2-deoxy-β-D-glucopyranosyl)-(1→3)-(2,4,6-tri-*O*-benzyl-β-D-galactopyranosyl)-(1→4)-2,3,6-tri-*O*-benzyl-β-D-glucopyranoside (13). NaOMe (0.2 mL, 1 M in MeOH) was added to a solution of **11** (2.90 g, 2.09 mmol) dissolved in MeOH (45 mL) and the solution stirred for 2.5 h, neutralized with Dowex H^+, filtered and concentrated. The product was dissolved in EtOH (50 mL) and ethylenediamine (5 mL) and the reaction refluxed for 7 h, concentrated, coevaporated with toluene and dried. The residue was dissolved in pyridine (20 mL) and Ac_2O (6 mL), stirred for 80 min, diluted with toluene,

washed with water and $NaHCO_3$ (aq), dried, concentrated and gave, after purification by column chromatography (toluene-EtOAc 3:1), 3-azidopropyl (2-acetamido-3-*O*-acetyl-4,6-*O*-benzylidene-2-deoxy-β-D-glucopyranosyl)-(1→3)-(2,4,6-tri-*O*-benzyl-β-D-galactopyranosyl)-(1→4)-2,3,6-tri-*O*-benzyl-β-D-glucopyranoside (**12**, 2.52 g, 1.94 mmol, 93 %). $[\alpha]_D$ −35.1 (*c* 1.1, $CHCl_3$). ^{13}C NMR ($CDCl_3$): δ 20.8, 22.9 ($COCH_3$), 29.3 ($OCH_2CH_2CH_2N_3$), 48.4 ($OCH_2CH_2CH_2N_3$), 54.6, 66.5, 66.6, 68.3, 72.2, 73.3, 73.4, 73.5, 74.5, 75.0, 75.1, 75.1, 75.5, 76.3, 76.5, 78.7, 80.5, 81.8, 83.0 (C-2^{I}-6^{I}, 2^{II}-6^{II}, 2^{III}-6^{III}, $OCH_2CH_2CH_2N_3$, CH_2Ph), 100.1, 101.8, 102.4, 103.5 (C-1^{I}, 1^{II}, 1^{III}, *C*HPh), 126.2–129.5, 138.2, 138.4, 138.7, 139.0, 139.1, 139.2 (aromatic *C*), 169.8, 171.1 ($COCH_3$, $NHCOCH_3$). A solution of **12** (1.22 g, 940 μmol) in THF (14 mL) was stirred together with 3Å molecular sieves for 30 min. $NaBH_3CN$ (301 mg, 4.78 mmol) was added, the mixture stirred for another 10 min followed by slow addition of HCl/Et_2O until no starting material could be detected by TLC. The reaction mixture was diluted with toluene (30 mL), filtered, washed with water and $NaHCO_3$ (aq), dried, concentrated and purified by column chromatography (toluene-EtOAc 3:1) to give **13** (990 mg, 761 μmol, 81%). $[\alpha]_D$ −21.7 (*c* 1.2, $CHCl_3$). ^{13}C NMR ($CDCl_3$): δ 20.9 ($COCH_3$), 22.9 ($NHCOCH_3$), 29.3 ($OCH_2CH_2CH_2N_3$), 48.4 ($OCH_2CH_2CH_2N_3$), 53.9, 66.5, 68.2, 70.7, 71.0, 73.4, 73.5, 73.9, 74.0, 74.5, 75.0, 75.2, 75.5, 75.9, 76.3, 76.5, 80.3, 81.3, 81.7, 82.9 (C-2^{I}-6^{I}, 2^{II}-6^{II}, 2^{III}-6^{III}, $OCH_2CH_2CH_2N_3$, CH_2Ph), 102.4, 102.7, 103.6 (C-1^{I}, 1^{II}, 1^{III}), 126.2-129.1, 137.5, 138.3, 138.4, 138.7, 139.1, 139.1, 139.3 (aromatic *C*), 169.8, 171.8 ($COCH_3$, $NHCOCH_3$). Anal. Calcd for $C_{74}H_{84}N_4O_{17}$: C, 68.29; H, 6.51. Found: C, 68.31; H, 6.57.

3-Azidopropyl (2,3-di-*O*-acetyl-4-*O*-benzyl-6-*O*-*tert*-butyldimethylsilyl-β-D-galactopyranosyl)-(1→4)-(2-acetamido-3-*O*-acetyl-6-*O*-benzyl-2-deoxy-β-D-glucopyranosyl)-(1→3)-(2,4,6-tri-*O*-benzyl-β-D-galactopyranosyl)-(1→4)-2,3,6-tri-*O*-benzyl-β-D-glucopyranoside (14). A solution of **3** (155 mg, 303 μmol) and **13** (210 mg, 161 μm) in CH_2Cl_2 (2.5 mL) containing 4Å molecular sieves was stirred for 1.5 h. The reaction mixture was cooled to −35 °C, NIS (157 mg, 697 μmol) added, stirred for another 10 min followed by addition of a catalytic amount of AgOTf. After 40 min at −25 °C, the reaction was quenched with triethylamine (0.5 mL), diluted with toluene, filtered through Celite, concentrated and purified by column chromatography (toluene-EtOAc 3:1) to afford **14** (217 mg, 124 μmol, 77%). $[\alpha]_D$ −6.4 (*c* 1.6, $CHCl_3$). ^{13}C NMR ($CDCl_3$): δ −5.4, −5.3 (Si*C*H_3), 18.2 (*C*$(CH_3)_3$), 20.8, 20.9 (CO*C*H_3), 22.8 (NHCO*C*H_3), 25.9 (C(*C*$H_3)_3$), 29.6 (OCH_2*C*$H_2CH_2N_3$), 48.4 (OCH_2CH_2*C*H_2N_3), 54.0, 60.1, 66.5, 68.1, 68.4, 70.6, 73.4, 73.4, 73.8, 73.8, 74.5, 74.6, 74.9, 75.0, 75.1, 76.6, 80.0, 81.7, 81.8, 82.9 (C-2^{I}-6^{I}, 2^{II}-6^{II}, 2^{III}-6^{III}, 2^{IV}-6^{IV}, O*C*$H_2CH_2CH_2N_3$, *C*H_2Ph), 100.6, 102.7, 102.8, 103.5 (C-1^{I}, 1^{II}, 1^{III}, 1^{IV}), 127.7-139.1 (aromatic *C*), 169.3, 169.9, 170.5, 170.9 (*C*OCH_3, NH*C*OCH_3). Anal. Calcd for $C_{97}H_{118}N_4O_{24}Si$: C, 67.79; H, 6.83. Found: C, 67.64; H, 6.76.

3-(*N*-Benzyloxycarbonyl)-aminopropyl (2-azido-3,4-*O*-dibenzyl-6-*tert*-butyldimethylsilyl-2-deoxy-α-D-galactopyranosyl)-(1→6)-(2,3-di-*O*-acetyl-4-*O*-benzyl-β-D-galactopyranosyl)-(1→4)-(3-*O*-acetyl-6-*O*-benzyl-2-acetamido-2-deoxy-β-D-glucopyranosyl)-(1→3)-(2,4,6-tri-*O*-benzyl-β-D-galactopyranosyl)-(1→4)-2,3,6-tri-*O*-benzyl-β-D-glucopyranoside (17). H_2S was bubbled through a solution of **14** (105 mg, 60 μmol) in pyridine (4 mL) and triethylamine (2 mL) for 7 h

and the solution was concentrated. Pyridine (0.2 mL) and benzylchloroformate (10 μl, 71 μmol) were added to a solution of the residue in CH_2Cl_2 (4 mL) at 0 °C, stirred for 20 min, concentrated and coevaporated with toluene. Purification by column chromatography (toluene-EtOAc 2:1) yielded 3-(*N*-benzyloxycarbonyl)-aminopropyl (2,3-di-*O*-acetyl-4-*O*-benzyl-6-*O*-*tert*-butyldimethylsilyl-β-D-galactopyranosyl)-(1→4)-(2-acetamido-3-*O*-acetyl-6-*O*-benzyl-2-deoxy-β-D-glucopyranosyl)-(1→3)-(2,4,6-tri-*O*-benzyl-β-D-galactopyranosyl)-(1→4)-2,3,6-tri-*O*-benzyl-β-D-glucopyranoside (**15**, 102 mg, 55 μmol, 91%). $[\alpha]_D$ −12.3 (*c* 2.4, $CHCl_3$). ^{13}C NMR ($CDCl_3$): δ −5.4, −5.3 (Si*C*H_3), 18.2 (*C*$(CH_3)_3$), 20.8, 20.9, (CO*C*H_3), 22.8 (NHCO*C*H_3), 25.9 (C(*C*H_3)$_3$), 29.7 (OCH_2*C*H_2CH_2NH), 38.2 (OCH_2CH_2*C*H_2NH), 54.0, 60.1, 66.6, 67.4, 68.1, 68.4, 70.6, 73.2, 73.4, 73.4, 73.8, 74.5, 74.7, 74.7, 74.9, 75.0, 75.1, 75.1, 75.4, 76.5, 76.6, 79.9, 81.7, 81.9, 82.9 (C-2^{I}-6^{I}, 2^{II}-6^{II}, 2^{III}-6^{III}, 2^{IV}-6^{IV}, O*C*$H_2CH_2CH_2$NH, *C*H_2Ph), 100.6, 102.7, 102.8, 103.5 (C-1^{I}, 1^{II}, 1^{III}, 1^{IV}), 127.8-128.4, 136.8, 137.9, 138.0, 138.3, 138.4, 138.7, 139.1, 139.3 (aromatic *C*), 156.5 156.6 (NH*C*OOCH_2Ph), 169.4, 169.9, 170.4, 171.3 (*C*OCH_3, NH*C*OCH_3). 1 M TBAF (130 μl) as a solution in THF was added to a solution of **15** (102 mg, 55 μmol) in THF (8 mL) and the reaction stirred for 24 h at 0 °C. The solution was diluted with toluene (5 mL), washed with 25 mM HCl (5 mL) and water, dried and concentrated. Purification by column chromatography (toluene-EtOAc 3:1) gave 3-(*N*-benzyloxycarbonyl)-aminopropyl (2,3-di-*O*-acetyl-4-*O*-benzyl-β-D-galactopyranosyl)-(1→4)-(-2-acetamido-3-*O*-acetyl-6-*O*-benzyl-2-deoxy-β-D-glucopyranosyl)-(1→3)-(2,4,6-tri-*O*-benzyl-β-D-galactopyranosyl)-(1→4)-2,3,6-tri-*O*-benzyl-β-D-glucopyranoside (**16**, 102 mg, 49 μmol, 89%). $[\alpha]_D$ −14.4 (*c* 1.0, $CHCl_3$). ^{13}C NMR ($CDCl_3$): δ 20.9 21.5 (CO*C*H_3), 22.8 (NHCO*C*H_3), 29.8

($OCH_2CH_2CH_2NH$), 38.2 ($OCH_2CH_2CH_2NH$), 55.9, 61.9, 66.6, 68.1, 68.2, 68.4, 70.3, 73.2, 73.4, 73.4, 73.7, 73.8, 73.9, 74.5, 74.7, 74.9, 75.0, 75.3, 75.4, 76.4, 76.6, 80.1, 81.6, 81.7, 82.9 (C-2^{I}-6^{I}, 2^{II}-6^{II}, 2^{III}-6^{III}, 2^{IV}-6^{IV}, $OCH_2CH_2CH_2NH$, CH_2Ph), 100.6, 102.6, 102.7, 103.5 (C-1^{I}, 1^{II}, 1^{III}, 1^{IV}), 127.8–128.4, 136.8, 137.5, 137.8, 137.9, 138.2, 138.3, 138.7, 139.0, 139.1, 139.2 (aromatic *C*), 156.7 ($NHCOOCH_2Ph$), 169.4, 170.0, 170.6, 171.0 ($COCH_3$, $NHCOCH_3$). Bromine was added to a cooled (0 °C) solution of **6** (76 mg, 111 μmol) in CH_2Cl_2 (2 mL). After 20 minutes the reaction mixture was concentrated, co-evaporated with toluene, dissolved in CH_2Cl_2 (0.5 mL) and added to a solution of acceptor **16** (99 mg, 53 μmol) in dry CH_2Cl_2 (1.3 mL) containing 4A molecular sieves. DMF (50 μl) and Et_4NBr (10 mg, 48 μmol) were added at 0 °C under a nitrogen atmosphere and the reaction was stirred at rt for 11 days. The mixture was diluted with toluene (10 mL), quenched with triethylamine (0.5 mL) and methanol (0.3 mL), filtered through a short silica column (toluene-EtOAc 3:1) and concentrated. The residue was dissolved in CH_2Cl_2 (8 mL) followed by addition of 0.1 μl $BF_3{\cdot}Et_2O$, stirred for 30 min, washed with water and $NaHCO_3$ (aq.), dried, concentrated and purified by column chromatography (toluene-EtOAc 3:1) to yield **17** (99 mg, 42 μmol, 79%). $[\alpha]_D$ +4.2 (*c* 1.2, $CHCl_3$). ^{13}C NMR ($CDCl_3$): δ 19.3 ($C(CH_3)_3$), 20.8, 20.9 ($COCH_3$), 22.9 ($NHCOCH_3$), 27.0 ($C(CH_3)_3$), 29.6 ($OCH_2CH_2CH_2NH$), 38.2 ($OCH_2CH_2CH_2NH$), 54.0, 60.0, 62.0, 65.3, 66.6, 67.4, 68.0, 68.1, 68.3, 68.4, 70.5, 71.4, 72.3, 72.6, 73.2, 73.5, 73.6, 73.9, 74.5, 74.9, 75.1, 75.5, 76.6, 77.8, 80.0, 81.7, 82.9 (C-2^{I}-6^{I}, 2^{II}-6^{II}, 2^{III}-6^{III}, 2^{IV}-6^{IV}, 2^{V}-6^{V}, $OCH_2CH_2CH_2NH$, CH_2Ph), 98.8, 100.7, 102.7, 102.9, 103.5 (C-1^{I}, 1^{II}, 1^{III}, 1^{IV}, 1^{V}), 127.8–135.6.1, 136.8, 137.6, 137.9, 138.3, 138.3, 138.4, 138.7, 139.0, 139.0, 139.3 (aromatic *C*), 156.5

(NHCOOCH$_2$Ph), 169.3, 169.6, 170.4, 170.8 (COCH$_3$, NHCOCH$_3$). Anal. Calcd for $C_{135}H_{151}N_5O_{30}Si$: C, 68.95; H, 6.47. Found: C, 68.90; H, 6.38.

3-(*N*-Benzyloxycarbonyl)-aminopropyl (2-acetamido-3,4-*O*-dibenzyl-2-deoxy-α-D-galactopyranosyl)-(1→6)-(2,3-di-*O*-acetyl-4-*O*-benzyl-β-D-galactopyranosyl)-(1→4)-(3-*O*-acetyl-6-*O*-benzyl-2-acetamido-2-deoxy-β-D-glucopyranosyl)-(1→3)-(2,4,6-tri-*O*-benzyl-β-D-galactopyranosyl)-(1→4)-2,3,6-tri-*O*-benzyl-β-D-glucopyranoside (19). H_2S was bubbled through a solution of **17** (105 mg, 60 μmol) in pyridine (4 mL) and triethylamine (2 mL) for 7 h and concentrated. Pyridine (0.5 mL) and Ac_2O (0.1 mL) were added to a solution of the residue in CH_2Cl_2 (2 mL) and stirred for 10 min. The reaction solution was diluted with toluene (3 mL), washed with water, dried and concentrated. Purification by column chromatography (toluene-EtOAc 2:1) gave 3-(*N*-benzyloxycarbonyl)-aminopropyl (2-acetamido-3,4-*O*-dibenzyl-6-*tert*-butyldimethylsilyl-2-deoxy-α-D-galactopyranosyl)-(1→6)-(2,3-di-*O*-acetyl-4-*O*-benzyl-β-D-galactopyranosyl)-(1→4)-(3-*O*-acetyl-6-*O*-benzyl-2-acetamido-2-deoxy-β-D-glucopyranosyl)-(1→3)-(2,4,6-tri-*O*-benzyl-β-D-galactopyranosyl)-(1→4)-2,3,6-tri-*O*-benzyl-β-D-glucopyranoside (**18**, 102 mg, 55 μmol, 91%). ^{13}C NMR ($CDCl_3$): δ 19.3 (C(CH$_3$)$_3$), 20.9 (COCH$_3$), 22.9, 23.4 (COCH$_3$), 27.0 (C(CH$_3$)$_3$), 29.6 (OCH$_2$$CH_2CH_2$NH), 38.2 (OCH$_2CH_2$$CH_2$NH), 49.2, 53.7, 62.6, 65.1, 66.6, 67.4, 68.0, 68.1, 68.3, 68.4, 70.0, 71.6, 72.4, 72.6, 73.2, 73.4, 73.5, 73.8, 74.0, 74.5, 74.6, 74.8, 74.9, 75.1, 75.5, 76.4, 76.6, 80.1, 81.7, 81.8, 82.9 (C-2^{I}-6^{I}, 2^{II}-6^{II}, 2^{III}-6^{III}, 2^{IV}-6^{IV}, 2^{V}-6^{V}, OCH$_2$CH$_2$CH$_2$NH, CH$_2$Ph), 97.9, 100.9, 102.7, 102.9, 103.5 (C-1^{I}, 1^{II}, 1^{III}, 1^{IV}, 1^{V}), 127.8–139.1 (aromatic C), 156.5 (NHCOOCH$_2$Ph), 169.3, 169.6, 170.0, 170.5, 170.9 (COCH$_3$, NHCOCH$_3$). 1 M

TBAF (45 μL) as a solution in THF was added to a solution of **18** (55 mg, 23 μmol) in THF (5 mL) and the solution stirred for 24 h at 0 °C, diluted with toluene (5 mL), washed with 15 mM HCl (3 mL) and water, dried and concentrated. Purification by column chromatography (toluene-EtOAc 1:1) produced **19** (42 mg, 20 μmol, 85%). $[\alpha]_D$ +10 (*c* 0.6, $CHCl_3$). ^{13}C NMR ($CDCl_3$): δ 20.9 ($COCH_3$), 22.8, 23.5 ($NHCOCH_3$), 29.7 ($OCH_2CH_2CH_2NH$), 38.3 ($OCH_2CH_2CH_2NH$), 49.2, 53.8, 61.7, 66.6, 67.4, 68.1, 68.4, 70.1, 71.3, 71.7, 72.3, 73.3, 73.4, 73.5, 73.8, 74.2, 74.3, 74.5, 74.9, 75.1, 75.3, 75.5, 76.4, 76.6, 77.7, 78.4, 80.1, 81.6, 81.7, 82.9 (C-2^{I}-6^{I}, 2^{II}-6^{II}, 2^{III}-6^{III}, 2^{IV}-6^{IV}, 2^{V}-6^{V}, $OCH_2CH_2CH_2NH$, CH_2Ph), 99.0, 100.6, 102.7, 102.8, 103.5 (C-1^{I}, 1^{II}, 1^{III}, 1^{IV}, 1^{V}), 127.8–128.9, 136.8, 137.5, 137.9, 138.2, 138.3, 138.5, 138.7, 139.0, 139.1, 139.2 (aromatic *C*), 156.6 ($NHCOOCH_2Ph$), 169.3, 170.0, 170.2, 170.8, 170.9 ($COCH_3$, $NHCOCH_3$). Anal. Calcd for $C_{121}H_{137}N_3O_{31}$: C, 68.25; H, 6.48. Found: C, 68.12; H, 6.41.

3-Aminopropyl (2-acetamido-2-deoxy-α-D-galactopyranosyl)-(1→6)-(β-D-galactopyranosyl)-(1→4)-(2-acetamido-2-deoxy-β-D-glucopyranosyl)-(1→3)-(β-D-galactopyranosyl)-(1→4)-β-D-glucopyranoside (20). 1 M NaOMe (0.1 mL) was added to a solution of **19** (41 mg, 19 mmol) dissolved in MeOH (3 mL). After 2.5 h of stirring the solution was neutralized with Dowex H^+, filtered, concentrated and purified by column chromatography (toluene-EtOAc 1:1) to afford 3-(*N*-benzyloxycarbonyl)-aminopropyl (2-acetamido-3,4-di-*O*-benzyl-2-deoxy-α-D-galactopyranosyl)-(1→6)-(4-*O*-benzyl-β-D-galactopyranosyl)-(1→4)-(2-acetamido-6-*O*-benzyl-2-deoxy-β-D-glucopyranosyl)-(1→3)-(2,4,6-tri-*O*-benzyl-β-D-galactopyranosyl)-(1→4)-2,3,6-tri-*O*-benzyl-β-D-glucopyranoside (33 mg, 16 μmol,

84%). ^{13}C NMR ($CDCl_3$): δ 23.1, 23.5 (NHCO*C*H_3), 29.7 (OCH_2*C*H_2CH_2NH), 38.3 (OCH_2CH_2*C*H_2NH), 49.3, 55.7, 62.2, 66.6, 67.4, 67.6, 68.1, 68.3, 69.9, 70.9, 71.5, 71.7, 72.8, , 73.4, 73.4, 73.5, 73.6, 73.7, 74.4, 74.4, 74.6, 74.9, 75.0, 75.1, 75.3, 75.4, 75.9, 76.4, 76.4, 76.5, 77.8, 77.8, 77.9, 80.4, 81.1, 81.7, 82.7, 82.9 (C-2^{I}-6^{I}, 2^{II}-6^{II}, 2^{III}-6^{III}, 2^{IV}-6^{IV}, 2^{V}-6^{V}, O*C*$H_2CH_2CH_2$NH, *C*H_2Ph), 98.0, 102.4, 102.7, 103.5, 104.0 5 (C-1^{I}, 1^{II}, 1^{III}, 1^{IV}, 1^{V}), 126.1–139.0 (aromatic *C*), 156.6 (NH*C*OOCH_2Ph), 170.2, 170.3 (NH*C*OCH_3). The deacetylated compound (22 mg, 11 μmol) was dissolved in MeOH (2 mL) and H_2O (0.5 mL) followed by addition of 1 M HCl (50 μL) and a catalytic amount of Pd (10% on C). The mixture was stirred under a H_2 atmosphere for 1 h, filtered, concentrated and purified through a C-18 (H_2O → MeOH) column to give **20** (7 mg, 7 μmol, 66%). $[\alpha]_D$ +29 (*c* 0.7, H_2O). ^{1}H NMR (D_2O) (selected data): δ 2.01 (m, 2H, CH_2 3-aminopropyl), 2.05, 2.05 (2s, 6H, NHAc), 3.16 (t, 2H), 3.33 (t, 1H), 4.16 (d, 1H), 4.43, 4.50, 4.51, 4.72 (4d, 4H, H-1^{I}, 1^{II}, 1^{III}, 1^{IV}) 4.92 (d, 1H , H-1^{V}). MALDI-TOF MS: Calcd for $C_{37}H_{65}N_3O_{26}$ ($[M+Na]^+$): 990.38, found 991.12. HRMS: Calcd for $C_{37}H_{66}N_3O_{26}$ ($[M+H]^+$): 968.3934, found 968,3943.

Triethylammonium 2-*tert*-butyloxycarbonylaminoethylphosphonate (21). Phosphorus trichloride (1.77 mL, 20.5 mmol) was added to a solution of imidazole (4.12 g) in dry MeCN (50 mL) at 0 °C. After 20 min of stirring at 0 °C, triethylamine was added followed by slow addition of a solution of N-Boc-ethanolamine (1.10 g, 6.83 mmol) in MeCN (10 mL). The reaction was stirred for 1 h at 0 °C, water (3 mL) added and the mixture partitioned between CH_2Cl_2 and water. The aqueous phase was washed with CH_2Cl_2-butanol 8:1 (3 × 50 mL) and the organic phase concentrated and coevaporated with toluene. The residue was purified by column chromatography

(CH_2Cl_2-MeOH-Et_3N 80:18:2) to yield **21** (842 mg, 2.17 mmol, 32%). ^{1}H NMR ($CDCl_3$): δ 1.04 (t, 9H, NCH_2CH_3), 1.12 (s, 9H, $C(CH_3)_3$), 2.79 (q, 6H, NCH_2CH_3), 3.01, 3.60 (2m, 4H, (OCH_2CH_2NH). ^{13}C NMR ($CDCl_3$): δ 8.1 (NCH_2CH_3), 27.9 ($C(CH_3)_3$), 45.2 (NCH_2CH_3), 61.9, 62.8 (OCH_2CH_2NH), 78.3 ($C(CH_3)_3$), 155.7 (NH*C*OOC).

3-Aminopropyl (2-acetamido-deoxy-6-*O*-(2-*tert*-butyloxycarbonylaminoethyl)phosphoryl-α-D-galactopyranosyl)-(1→6)-(β-D-galactopyranosyl)-(1→4)-(2-acetamido-2-deoxy-β-D-glucopyranosyl)-(1→3)-(β-D-galactopyranosyl)-(1→4)-β-D-glucopyranoside (23). Compound **19** (20 mg, 9 μmol) and phosphonate **21** (8 mg, 25 μmol) were concentrated from pyridine and dried under vacuum. The residue was dissolved in MeCN (1.5 mL) and pyridine (0.5 mL), cooled to 0 °C, pivaloyl chloride (3 μL, 24 μmol) added and the solution stirred for 4 h. The solution was diluted with toluene (5 mL), washed with water, dried and concentrated. The residue was dissolved in pyridine (1.5 mL), cooled to 0 °C, water (10 μl) and iodine (14 mg, 55 μmol) were added. The solution was stirred for 2.5 h, diluted with EtOAc (6 mL), washed with $Na_2S_2O_3$ (aq). Purification by column chromatography (CH_2Cl_2-MeOH-Et_3N 95:5:1) gave the triethylammonium salt of 3-(*N*-benzyloxycarbonylamino)propyl (2-acetamido-3,4-*O*-dibenzyl-2-deoxy-6-*O*-(2-*tert*-butyloxycarbonylaminoethyl)phosphoryl-α-D-galactopyranosyl)-(1→6)-(2,3-di-*O*-acetyl-4-*O*-benzyl-β-D-galactopyranosyl)-(1→4)-(3-*O*-acetyl-6-*O*-benzyl-2-acetamido-2-deoxy-β-D-glucopyranosyl)-(1→3)-(2,4,6-tri-*O*-benzyl-β-D-galactopyranosyl)-(1→4)-2,3,6-tri-*O*-benzyl-β-D-glucopyranoside (**22**). MALDI-TOF MS: Calcd for $C_{128}H_{151}N_4O_{36}P$ ($[M+Na]^+$): 2375.54, found 2375.91. HRMS: Calcd

for $C_{128}H_{150}N_4O_{36}P^-$ ($[M-H]^-$): 2349.9768, found 2349.9729. 1 M NaOMe (50 μL, in MeOH) was added to a solution of **22** dissolved in MeOH. After 6 h of stirring the solution was neutralized with dry ice, concentrated and purified by column chromatography (CH_2Cl_2-MeOH-Et_3N 90:10:1) to give the deacetylated compound. MALDI-TOF MS: Calcd for $C_{122}H_{145}N_4O_{33}P$ ($[M+2Na]^+$): 2272.42, found 2271.89. A catalytic amount of $Pd(OH)_2$ (20% on C) was added to a solution of the product from the last step in MeOH (3 mL) and H_2O (1.2 mL). The mixture was stirred under a H_2 atmosphere for 5 h, filtered and concentrated. The residue was purified through a C-18 column (H_2O → MeOH) yielding **23** (5 mg, 4 μmol, 44%). $[\alpha]_D$ +34.5 (*c* 0.2, H_2O). ^{1}H NMR (D_2O) (selected data): δ 1.41 (s, 9H, CH_3 Boc), 1.97 (m, 2H, CH_2 3-aminopropyl), 2.02, 2.02 (2s, 6H, NHAc), 3.13 (t, 2H), 3.30 (t, 2H), 3.31 (t, 1H), 4.13 (d, 1H), 4.40, 4.45, 4.48, 4.69 (4d, 4H, H-1^{I}, 1^{II}, 1^{III}, 1^{IV}) 4.90 (d, 1H , H-1^{V}). ^{31}P NMR (D_2O): δ 1.02. MALDI-TOF MS: Calcd for $C_{44}H_{78}N_4O_{31}P$ $[M+2Na]^+$: 1235.42, found 1236.73. HRMS: Calcd for $C_{44}H_{78}N_4O_{31}P$ $[M]^-$: 1189.4387, found 1189.4373.

Reaction of 20 with dimethyl squarate. Compound **20** (2.3 mg, 2.4 μmol) was dissolved in MeOH (800 μL) followed by addition of dimethyl squarate [67 μL, 2 equiv as a solution in MeOH (10 mg/mL)] and triethylamine [36 μL, 1.5 equiv, as a solution in MeOH (10 mg/mL)]. After 8 h, the reaction was complete according to MALDI-TOF and the solution concentrated and purified through a short C-18 column (H_2O → MeOH). MALDI-TOF MS: Calcd for $C_{42}H_{67}N_3O_{29}$ ($[M+Na]^+$): 1100.38, found 1101.30.

Reaction of 23 with dimethyl squarate and deprotection of Boc. Compound **23** (1.2 mg, 1.0 μmol) was dissolved in MeOH (600 μL) followed by addition of dimethyl squarate [22 μL, as a solution in MeOH (10 mg/mL)] and triethylamine [12 μL, as a solution in MeOH (10 mg/mL)]. After 4 h, the reaction was complete according to MALDI-TOF and the solution concentrated. The residue was dissolved in water (900 μL) and TFA (90 μL) added. The mixture was stirred for 5 h, when no *N*-Boc protected starting material could be detected by MALDI-TOF. The solution was concentrated, coevaporated with water and the crude product passed through a short C-18 column (H_2O → MeOH) to yield **24**.

HSA-conjugate of 20. Dimethylsquarate activated **20** (0.7 mg) was dissolved in 0.5 M sodium phosphate buffer (250 μL, pH 10) and a solution of HSA (95 μL, 10 mg/mL) was added. After 48 h, the reaction solution was filtered by centrifugation through a 30 kDa filter, the precipitate diluted with water and filtered once again. The procedure was repeated twice. The residue was diluted with water, filtered through a sterile filter and freeze dried. According to MALDI-TOF an incorporation of about 16 sugar residues per protein molecule was obtained.

HSA-conjugate of 24. A solution of HSA (50 μL, 10 mg/mL) was added to a solution of **24** (0.5 mg) in 0.5 M sodium phosphate buffer (200 μL, pH 10). After 72 h, the reaction solution was filtered by centrifugation through a 30 kDa filter, the precipitate diluted with water and filtered once again. The procedure was repeated twice. The

residue was diluted with water, filtered through a sterile filter and freeze dried. An incorporation of about 7 mol mol^{-1} was obtained.

Biotinylation of 23. (+)-Biotin *N*-hydroxy-succinimide ester (1.5 mg) and Et_3N (2 μL) were added to a 0.1 M pH 7 buffer solution (500μL) of **23** (0.5 mg). The reaction mixture was stirred for 20 min until no starting material could be detected by TLC (EtOAc-HOAc-MeOH-H_2O 2:3:3:2), filtered through a short C-18 column (H_2O → MeOH) and the combined carbohydrate-containing fractions were concentrated. The residue was dissolved in H_2O (300 μL) and TFA (60 μL) and the solution stirred for 3 h, concentrated and the residue purified through a short C-18 column to afford **25**. MALDI-TOF MS: Calcd for $C_{49}H_{84}N_6O_{31}PS$ ($[M+H]^+$): 1317.47, found 1318.3.
